# Hydrological Analysis of Batu Dam, Malaysia in the Urban Area: Flood and Failure Analysis Preparing for Climate Change

**DOI:** 10.3390/ijerph192416530

**Published:** 2022-12-09

**Authors:** Siti Mariam Allias Omar, Wan Noorul Hafilah Wan Ariffin, Lariyah Mohd Sidek, Hidayah Basri, Mohd Hazri Moh Khambali, Ali Najah Ahmed

**Affiliations:** 1 Sustainable Technology and Environment Group, Institute of Energy Infrastructure, Universiti Tenaga Nasional (UNITEN), Kajang 43000, Malaysia; 2 Dam and Design Division, Drainage and Irrigation Department, Kuala Lumpur 50480, Malaysia

**Keywords:** PMP, PMF, flood mitigation, Batu Dam in Malaysia, energy

## Abstract

Extensive hydrological analysis is carried out to estimate floods for the Batu Dam, a hydropower dam located in the urban area upstream of Kuala Lumpur, Malaysia. The study demonstrates the operational state and reliability of the dam structure based on hydrologic assessment of the dam. The surrounding area is affected by heavy rainfall and climate change every year, which increases the probability of flooding and threatens a dense population downstream of the dam. This study evaluates the adequacy of dam spillways by considering the latest Probable Maximum Precipitation (PMP) and Probable Maximum Flood (PMF) values of the concerned dams. In this study, the PMP estimations are applied using comparison of both statistical method by Hershfield and National Hydraulic Research Institute of Malaysia (NAHRIM) Envelope Curve as input for PMF establishments. Since the PMF is derived from the PMP values, the highest design flood standard can be applied to any dam, ensuring inflow into the reservoirs and limiting the risk of dam structural failure. Hydrologic modeling using HEC-HMS provides PMF values for the Batu dam. Based on the results, Batu Dam is found to have 200.6 m^3^/s spillway discharge capacities. Under PMF conditions, the Batu dam will not face overtopping since the peak outflow of the reservoir level is still below the crest level of the dam.

## 1. Introduction

A dam can be considered a large dam if its height from the foundation is 15 m or higher, or between 5 and 15 m, and its storage capacity is larger than three million cubic meters as defined by the International Commission on Large Dams (ICOLD) [1]. Large dam failure raises concerns in many nations because of the serious economic and social consequences that might result from it [2]. The hydrological regime may alter the dam’s usual condition as a result of climate change and runoff will likely shift significantly in the 21st century as a result of changing rainfall patterns and rising temperatures [3]. In common usage, the term “hydrology” now refers to research on precipitation and runoff that has been connected to issues with the design and management of water resource projects including dam functioning as flood control and water supply [4]. 

Traditional dam design methods concentrate on deterministic consideration of severe occurrences, such as the probable maximum flood (PMF). The PMF is the maximum amount of flooding a logically possible set of meteorological and hydrologic circumstances might possibly expect. In other words, PMF assumes the dependability of zero failure while taking into account the maximum range of flood potential [5]. PMF is usually estimated by using Probable Maximum Precipitation, which also known as PMP using as an input in hydrologic modeling software.

The PMP over the study region is needed to calculate the PMF against the dam. There are several methods available for PMP estimation which can be categorized as hydrometeorological and statistical [6,7,8]. The meteorological approach is based on maximization of atmospheric moisture content during intense storm conditions, whereas the statistical approach is based on frequency-based statistical analysis and is computed using maximum rainfall occurred over historical periods and standard deviation of annual maximum rainfall [8]. The moisture maximization method, storm transposition method, generalized method, storm separation method, and depth-area duration method are all common hydrometeorological methods. The Hershfield method and its variants, as well as the multifractal approach, are common statistical methods [7]. It has the advantages of taking real historical data in the area of interest into consideration, describing it in terms of statistical characteristics, and being simple to apply [8,9].

The common potential failure mode for the dam will be overtopping. In order to prevent overtop at the dam crest, a study is conducted for the hydrological evaluation to determine if the dam has the spillway capacity to safely manage the PMF. This typical method for calculating the hydrological risk of dam overtopping is based on a univariate analysis that only takes into account flood peak discharges from the most PMF [5,10,11].

In this paper, the study area will be the Batu Dam, which is located in the urban area of Kuala Lumpur in the country of Malaysia. The primary objective of this study is to perform hydrological analysis including rainfall time series and double mass curve to verify the reliability of the hydrological data. Then, an analysis of PMP is conducted by comparing two statistical methods of Hershfield’s [12,13] and by NAHRIM envelope curve specifically for Peninsular Malaysia [14], which then will be an input to estimate the PMF for dam safety check with the spillway design capacity to predict the probability of overtopping.

## 2. Materials and Methods

### 2.1. Study Area

In response to the disastrous flood that hit Kuala Lumpur in 1971, USBR (The United States Bureau of Reclamation) developed the flood control and water supply dam known as Batu Dam as part of the Kuala Lumpur Flood Mitigation (KLFM) Project. It began to be constructed in 1985 and was finished in 1987. Approximately 20 km north of Kuala Lumpur, the Batu Dam catchment is situated in Peninsular Malaysia at 3°16′20′′ N latitude and 101°41′9′′ E longitude. The Batu Dam location is near Gombak Selangor, about 16 km upstream of the Kuala Lumpur city center, and is situated just downstream of the meeting point of two rivers known locally as Sungai Batu and Sungai Tua [15].

The Batu dam is built on schist, with schistosity dipping downstream. This schist is weathered to varied degrees, ranging from entirely weathered to fresh. The foundation of the embankment’s core zone and upstream part is made of weathered and fresh schist, whereas the foundation of the embankment’s downstream section is made of alluvial gravel layer [16]. Batu Dam is located in state of Selangor, which has an equatorial climate that is heavily influenced by the East Asian Monsoon. Selangor typically has two rainy seasons every year, with the south west monsoon from June to September and the north monsoon from November to March [17,18]. The Batu Reservoir was constructed to be used as a water supply for municipal and industrial purposes, as sediment management, and to partially regulate flooding in Kuala Lumpur. It offers flood storage up to the 100-year frequency of flood. The construction of the dam cost RM 20 million (excluding land purchase). Flood in the downstream communities has been decreased by activating Batu Dam and raising the level of water in the reservoir. In order to supply various areas of Kuala Lumpur with household and industrial water, Puncak Niaga is now abstracting water from the dam [15].

**Figure 1:**
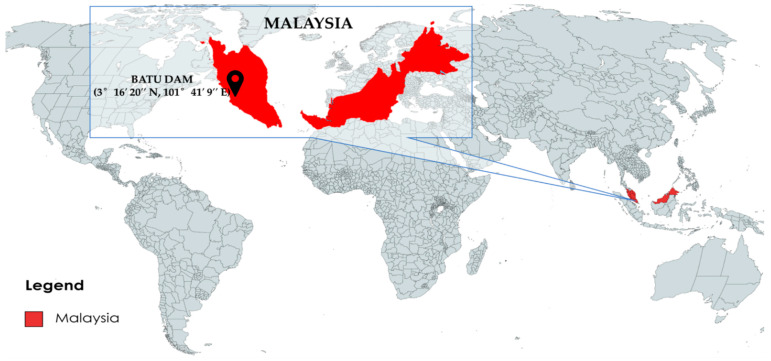
Location of Batu Dam, Malaysia in World Map.

**Figure 2:**
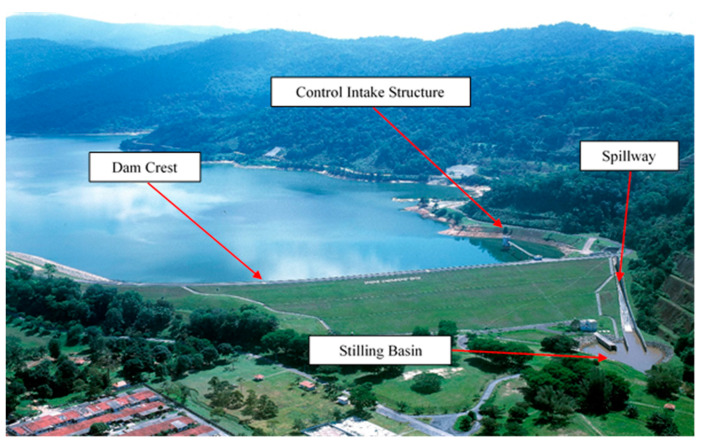
Components of Batu Dam.

An earthfill main embankment, a spillway, and outlet work make up the project’s primary components. The dam is an earthfill embankment that is zoned [16]. A side-channel entrance structure with a crest works as the spillway. The outlet works are situated at the base of the left abutment to the right of the spillway structure. Water is delivered by the outflow works to the control structure’s municipal and industrial pipeline [15]. The combined spillway and outlet work total surcharge of 251.6 m^3^/s is offered as insurance against the likely maximum flood (PMF) and 234.2 m^3^/s of spillway capacities when outlet works is malfunction. Figure 1 shows the location of the dam in world map. Figure 2 displays an overview of Batu Dam and its components while Table 1 shows Batu Dam’s salient features.

### 2.2. Methods

#### 2.2.1. Hydrological Analysis

1. Rainfall Time Series

Time-series analysis has evolved into an important technique in hydrology. It is used to construct synthetic hydrologic records, anticipate hydrologic occurrences, and discover patterns and shifts in hydrologic records [19].

2. Double Mass Curve (DMC)

DMC is used to assess the regularity of various hydrologic data by assessing data from one station of interest to a pattern made of data from different stations in the region [20]. Due to its minimal data approach and high transferability, the DMC is frequently used for trend analysis [21]. Any DMC breakdowns may be the result of modifications made to the method of data collection or to physical modifications that have an effect on the relationship [20].

3. Rainfall Temporal Pattern

For both rural and urban catchments, runoff routing methods or hydrologic models like unit hydrographs require rainfall temporal patterns in order to estimate floods [22]. In this study, the rainfall temporal pattern for Batu Dam catchment is available and published in Chapter 8 of Hydrological Procedures 1 (HP1) revised and updated 2015, which falls on Region 5–Urban Area (Kuala Lumpur) [23]. The patterns were available for 9 standard durations, 0.25, 0.5, 1, 3, 6, 12, 24, 48, and 72 h.

4. Probable Maximum Precipitation (PMP)

PMP is “the highest depth of precipitation for a given duration meteorologically achievable for a given size storm area at a certain place at a particular time of year” [23,24]. For the catchment of a dam, hydrologists utilize a PMP magnitude together with its geographical and temporal distributions to determine the likely maximum flood (PMF). In the construction of hydrological structures, a variety of conceptual flood events are utilized, including the PMF. It is mostly used to design spillways that decrease the probability of a dam overtop [14].

Hershfield developed the statistical method that is used to calculate the PMP rainfall for a specific place or region using a general frequency equation they established [25,26] There are statistical techniques for estimating PMP that may be applied whenever there is enough precipitation data [27]. These methods are especially helpful when doing rapid estimations or when additional meteorological data such as wind records and dew point are absent [14]. The statistical approach developed by Hershfield is frequently employed today [7,8,9,10]. 

For stations with lengthy rainfall records, the Hershfield’s statistical methods are frequently employed to estimate PMP. If the data are not long enough to capture the excessive rainfall, PMP values may be underestimated [28]. Therefore, since the daily rainfall records are available for the study area, it is thus deemed appropriate to conduct a study on the approximation of PMP for hourly (1-, 3-, 6-, and 12-h and daily (1-, 2-, 3-, and 5-day) duration using the Hershfield approach using the following formula:

(1) $$\text{Xpmp}={\bar{x}}_{n}+\text{Km}\cdot \sigma_{\text{n}}$$

where Km represents the frequency factor, σ_n_ is the series of standard deviations (SD), and ${\bar{x}}_{n}$ is the mean of the annual maximum precipitation. In order to make the decision, it is necessary to obtain the mean and SD of the series for each station without taking into account the 10 years’ worth of highest precipitation, where

(2) $${\text{K}}_{\text{m}}=\left({\text{X}}_{1}-{\bar{x}}_{\text{n}-1}\right)/\sigma_{\text{n}-1}$$

Hershfield discovered that if an adequate envelope curve could be built based on the research area’s locations, it would yield accurate estimates of the PMP for different time periods of rainfall [12,28]. In 2008, National Hydraulic Research Institute Malaysia (NAHRIM) established an envelope curve for both in West and East Malaysia as a standard method of estimating PMP in Malaysia [14]. 

**Figure 3:**
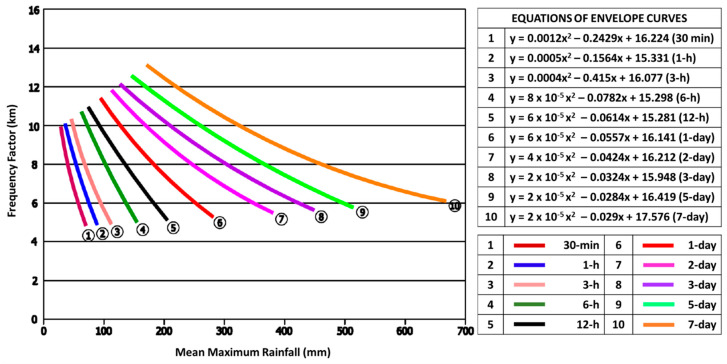
The Km envelope curve (West Malaysia) [14].

Figure 3 shows the Km envelope curve (west Malaysia), which is considered as an alternative method to calculate the Km frequency factor, which was developed by NAHRIM [14]. The PMP is computed using both Km frequency factors, and the most critical values from each approach are used as the input rainfall for the PMF simulation.

#### 2.2.2. Hydrological Modeling

1. Probable Maximum Flood (PMF)

The flood that could be predicted from the most acute combination of adverse meteorological and hydrologic circumstances is known as the PMF [29]. The PMF is produced using the probable maximum precipitation (PMP) input data for this research area [27].

**Figure 4:**
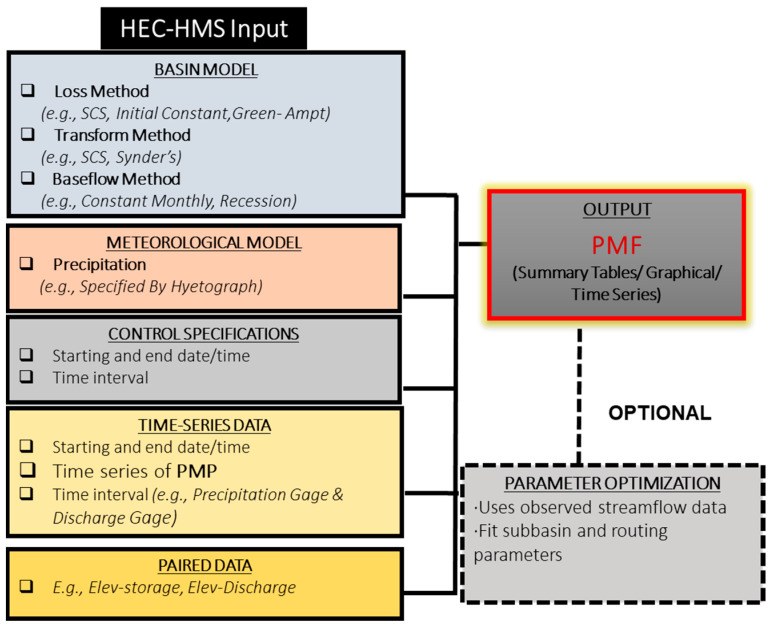
HEC-HMS Input.

**Figure 5:**
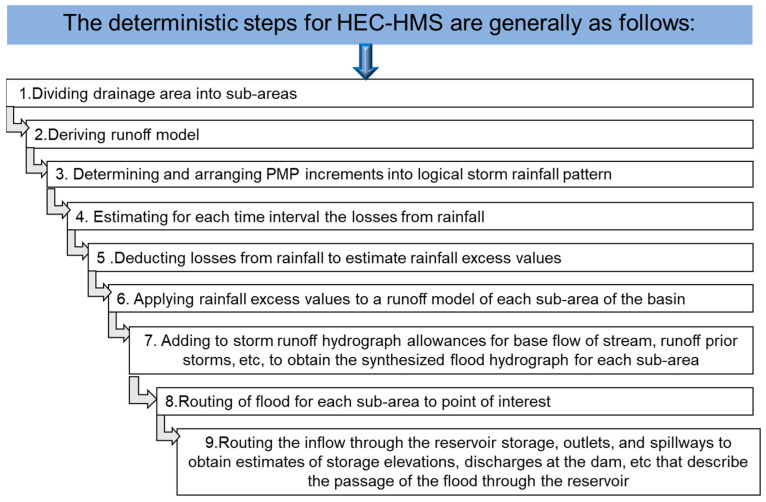
Steps for HEC-HMS simulation.

The Hydrologic Modeling System (HEC-HMS) software developed by USACE will be used to model the entire hydrologic process of branched watershed systems due to PMP to determine the PMF inflow of Batu Dam. For the HEC-HMS simulation, the required data are hydrological data, rainfall data, details of the dam, elevation-storage curve, and elevation-discharge curve. This model consists of several inputs such as basin model, meteorological model, control specification, time series data, and paired data as shown in Figure 4 [30]. Then, the deterministic steps for HEC-HMS simulation are illustrated in Figure 5 [29].

2. Delineation of Subcatchment

**Figure 6:**
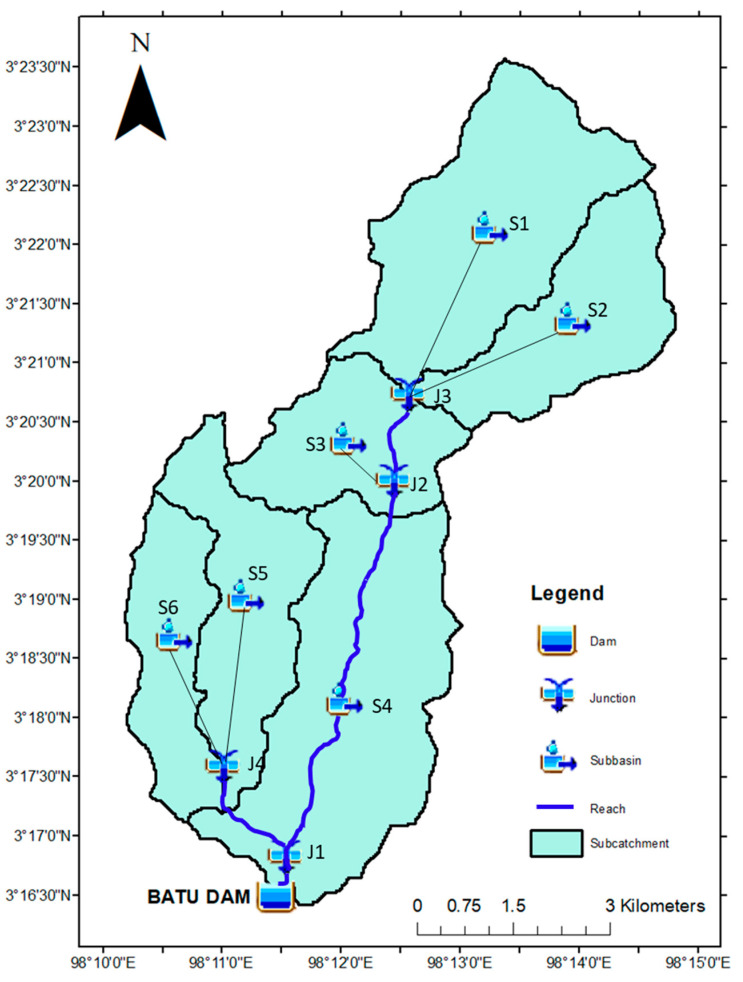
Basin Model Setup for Batu Dam Scheme.

The overall catchment area for Batu Dam is about 50 km^2^, which is then divided into six smaller subcatchments, namely S1–S6 characterized by different soil type and land use pattern delineated using GIS features in the HEC-HMS 4.8 model providing Digital Elevation Model (DEM) of ALOS PALSAR with resolution of 30 m in a coordinate system of Universal Transverse Mercator (UTM). The delineated six subcatchments are depicted in Figure 6. 

All physical characteristics of the Batu Dam subcatchments such as the area of subcatchments, the longest flow path, elevation, and slope of the river can be obtained from the GIS features in HEC-HMS model after completing the delineation process, while study by the authors of [31] still used the ArcGIS tool to determine the physical parameter of the catchment for the input in the HEC-HMS model. All these physical characteristics then will be used to determine the input parameters by using the Malaysia guideline from [32] and calibrated by using guidelines from [33] as tabulated in Table 2. 

## 3. Results and Discussion

### 3.1. Rainfall Time Series

**Figure 7:**
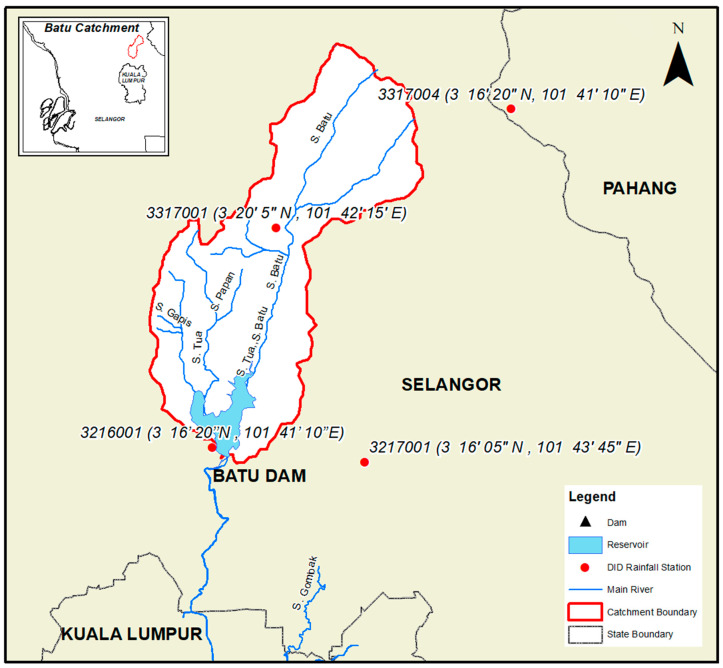
Rainfall stations within the Batu Dam catchment.

For this study, four selected rainfall stations that fall within the Batu Dam catchment are used for the hydrological analysis which are Kg Sg Tua (3216001), Ibu Bekalan KM16 (3217001), Air Terjun Sg Batu (3317001), and Genting Sempah (3317004) as shown in Figure 7.

**Figure 8:**
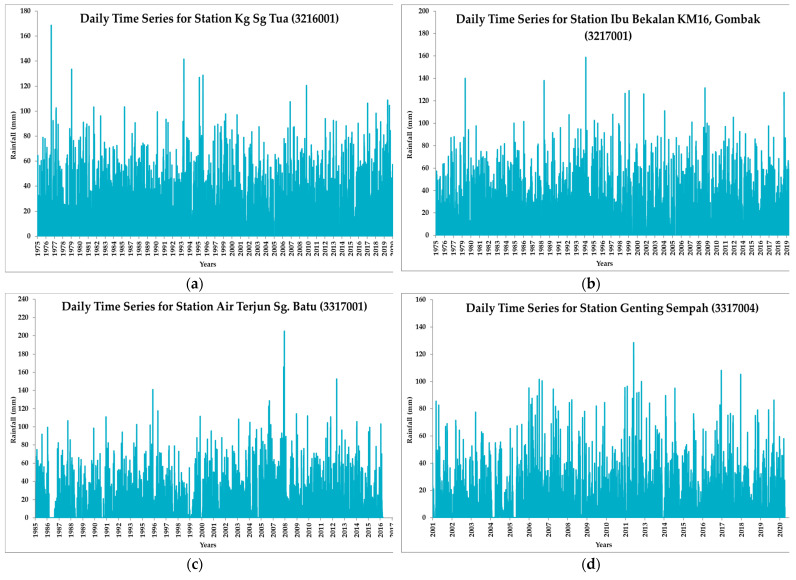
Daily rainfall time series of the station (**a**) Kg Sg Tua (3216001), (**b**) Ibu Bekalan KM16 (3217001), (**c**) Air Terjun Sg Batu (3317001), and (**d**) Genting Sempah (3317004).

From the plotted daily rainfall time series (Figure 8), it can be observed that the highest recorded rainfall in station Kg Sg Tua (3216001) is 168.5 mm on 16 October 1976, Ibu Bekalan KM16 (3217001) with 158.5mm on 27 June 1994, Air Terjun Sg. Batu (3317001) with 204.0 mm on 1 February 2008, and Genting Sempah (3317004) with 128.4 mm on 31 May 2012.

### 3.2. Double Mass Curve

**Figure 9:**
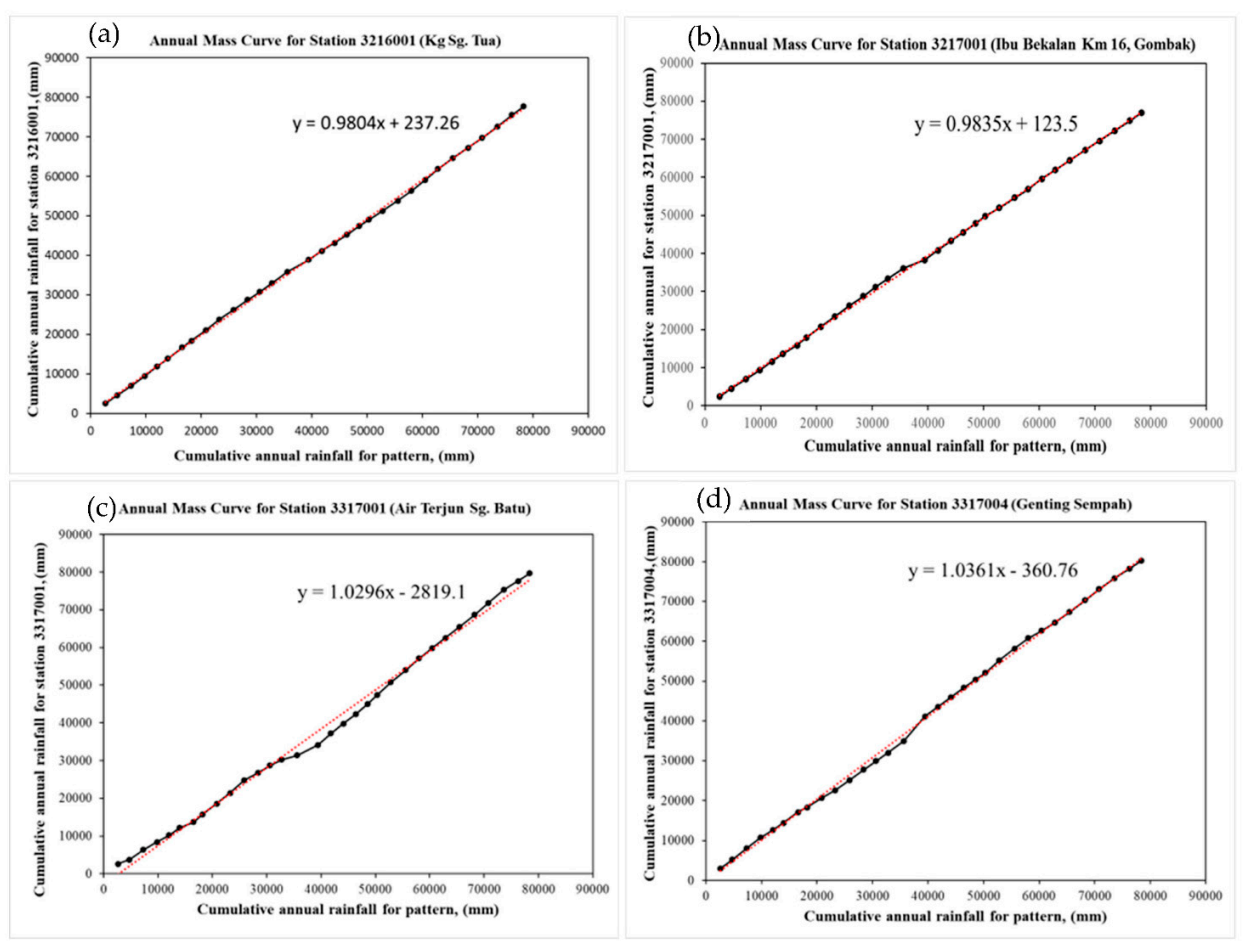
Double mass curve of the station (**a**) Kg Sg Tua (3216001), (**b**) Ibu Bekalan KM16 (3217001), (**c**) Air Terjun Sg Batu (3317001), and (**d**) Genting Sempah (3317004).

According to the plots (Figure 9), all four stations records show the consistency of data. The slope of all four mass curves with good consistency and quality as the slope value is close to 1 (Station No. 3216001: 0.99; Station No. 3217001: 0.99, Station No. 3317001: 1.03; and Station No. 3317004: 1.04).

### 3.3. Isohyet Map

**Figure 10:**
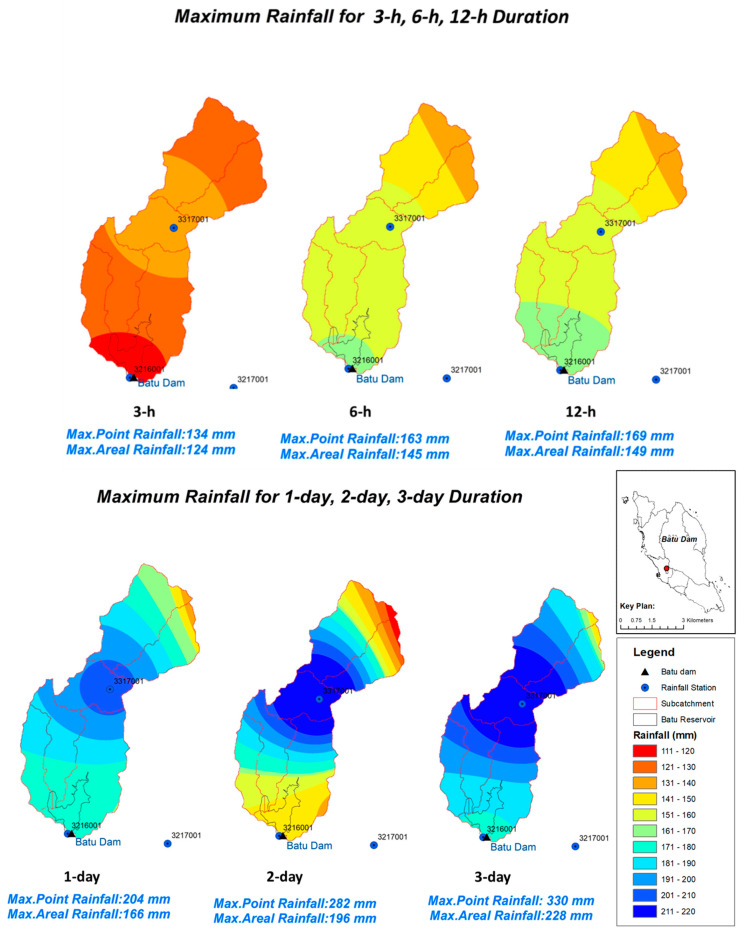
Isohyets Map for the recorded rainfall (extreme events) in mm different durations.

Based on the hydrological analysis, maximum rainfall for (3-, 6-, and 12-h) and (1-, 2-, and 3-day) was acquired from the historical data up to the latest year. The interpolation method from GIS analysis has been carried out to create an Isohyet map for the studied catchment. The isohyet method was used to split the area based on the rain intensity, with contours functioning as dividing lines for the differences in rainfall intensity [34]. Figure 10 shows the maximum rainfall intensity of the Batu Dam catchment for different durations.

Based on Figure 10, the rainfall intensity for 3-h and (1, 2, and 3-day) durations are high at the center of the catchment which is located at the rainfall station of Air Terjun Sg Batu (3317001). Based on Google Earth, it can be observed that the location of this rainfall station is located in a thick rainforest while the location of the dam is located near to population downstream. The highest received rainfall in the Batu Dam catchment was in the range of 201mm to 220mm for (1, 2, and 3-day) durations, which are located at the middle upstream, can cause the increase of reservoir water level as it flows into the reservoir. Thus, dam monitoring during heavy rainfall event is very crucial for us to estimate the Rate of Rise (ROR) of the dam in case an emergency Action Plan (EAP) need to be activated.

### 3.4. Probable Maximum Precipitation (PMP)

Using the statistical approach, PMP is predicted for the following time periods: (1, 3, 6, and 12-h) and (1, 2, 3, and 5-day). Table 3 displays the projected PMP for Batu Dam for these periods using both statistical method by Hershfield’s and NAHRIM. By comparing both methods, it is found that PMP values using NAHRIM envelope curve gives higher value than Hershfield’s method. Thus, by considering worst case scenario, PMP values derived by using NAHRIM method will be adopted in this study to estimate the PMF.

The approach that gives the greatest PMP values is chosen as the most preferable method to be employed as rainfall inputs for PMF analyses. Previous studies by [35,36] also found that based on these two methods of PMP estimation between Hershfield’s method of derived Km and the NAHRIM Km envelope curve, the NAHRIM envelope curved is found to be the most reliable since it gives the higher estimation of PMP.

### 3.5. Probable Maximum Flood (PMF) and Dam Safety Check

**Figure 11:**
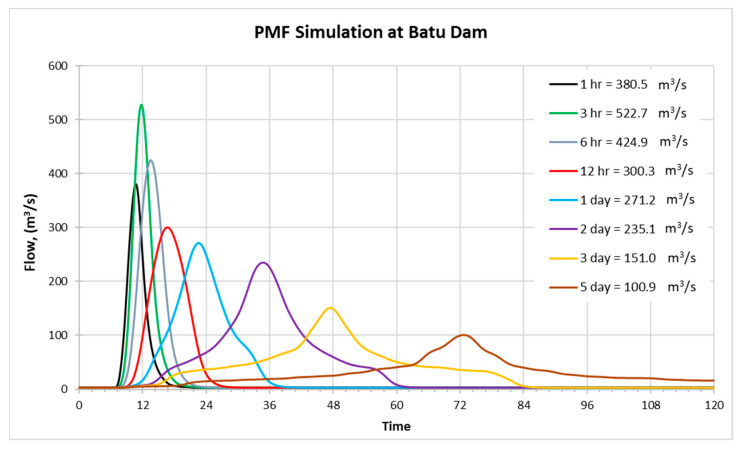
PMF simulations at Batu dam.

By using the HEC-HMS model, PMF is then simulated for the duration of (1-, 3-, 6-, and 12-h) and (1-, 2-, 3-, and 5-day). Plotted results of PMF simulation at Batu Dam for various durations can be referred to in Figure 11. From Figure 11, it is found that the extreme flood of Batu Dam peaked at 3-h duration with PMF inflow of 522.7 m^3^/s.

The dam’s designed inflow value is compared with the highest PMF value for the Batu dam. It was discovered that the PMF of Batu Dam is 6.1% above the designed inflow values. Since the modified design flood is based on recent standards as required by ICOLD, and the inflow design is calculated using the Inflow Design Flood (IDF), the value is reasonable and expected. Additionally, the spillway capacity for the Batu dam is discovered to be sufficient to resist the peak flood PMF.

The PMF simulated spillway capacity for the Batu dam is 200.6 m^3^/s. It was found that these values are 14.4% lower in comparison to the designed spillway capacities. The simulated reservoir level also found to be 0.6 m lower than the designed level. Table 4 compares the designed inflow and revised PMF for the Batu dam along with the designed spillway capacities and the simulated reservoir level.

Even though the inflow of PMF is higher than the design inflow, the design spillway capacity still can cater the revised spillway discharge. The simulated reservoir level is lower than the reservoir level obtained from designed inflow, implying that the simulated inflow volume from PMF in this study is lesser than the designed inflow volume. The discrepancy may be due to different initial dam condition boundary including adopted elevation-storage curve during the design stage and current simulation resulting in lower peak discharge compared to design capacities. It can be concluded that the existing spillway still can cater to the estimated inflow due to PMF which reduces the probability of overtopping with freeboard of 1.6 m from the dam crest.

## 4. Conclusions

Since the statistical method of estimating PMP require real historical rainfall data in the study area, hydrological analysis including rainfall time series and double mass curve allow us to discover the patterns of the historical rainfall data and reliability of the record. Computation of PMP and PMF are important in dam safety as it can be a simple safety check and suggestions on future dam failure criteria especially in hydrological type of failure mode. By comparing both statistical methods of computing PMP values by Hershfield’s derived Km and using the recent NAHRIM published envelope curve, it can be observed that PMP values by using the NAHRIM envelope curve are higher than the Hershfield’s derived Km method, which then has been adopted as input for the hydrological modeling to estimate the PMF. This finding seems to be concurrent with the statement by Hershfield [12,37] as if an envelope curve can be built based on the study area, it would yield accurate estimation of the PMP values. An established hydrologic modeling software by USACE, which is HEC-HMS, is then used to estimate the PMF value for Batu Dam as well as the simulated spillway discharge due to PMF inflow. The estimated PMF for the Batu dam is found to exceed the capacities of the design inflow. Nevertheless, under PMF conditions, the Batu dam will not face overtopping since the simulated reservoir level is still below the crest level of the dam. The spillway capacities of the Batu dam also seem to be adequate with the design capacities of 200.6 m^3^/s. Therefore, it is suggested to allocate additional facilities such as rainfall stations at the upstream catchment to observe the inflow into the catchment and streamflow stations at the downstream to observe the outflow of the catchment. It is also recommended to implement Risk Informed Decision Making (RIDM) for the Batu dam as a tool for dam safety assessment to minimize the impact of dam failure located in an urban area.

## Figures and Tables

**Table 1:** Salient features of Batu Dam.

BATU DAM	
Function	Water supply for both municipal and industrial, sediment control, and flood control
Direct Catchment Area	50 km^2^
Surface Area at Maximum FSL	2.5 km^2^
Normal Pool Level	El. 102.7 m
Full Supply Level	EL 107.3 m
RESERVOIR	
Storage Capacity	36.6 Mm^3^
Peak inflow design flood (IDF)	492.7 m^3^/s
Design Discharge Capacities	251.6/s (spillway and outlet) 234.2 m^3^/s (spillway only)
DAM	
Type	Earthfill
Height	44 m
Length	550 m
Crest Elevation (m)	El. 109.0 m
SPILLWAY	
Crest Elevation	El. 104.85 m
Invert Elevation	El. 97.83 m at upstream
Crest Length	23 m
Total length	226.53 m to the stilling basin

**Table 2:** Input Parameter of HEC-HMS.

Sub-Basin	Loss Method			Transform Method		Baseflow Method
	Initial and Constant			Clark (UH)		Constant Monthly
	Initial Loss (mm)	Constant Rate (mm/h)	Imperviousness (%)	SCS T_c_ Eq. (h)	R (h) HP27	HP 27 (m^3^/s)
S1	30	2.5	10	3.11	1.218	0.905
S2	30	2.5	10	2.66	1.116	0.584
S3	30	2.5	10	2.18	0.839	0.496
S4	30	2.5	10	6.03	2.262	1.073
S5	30	2.5	10	4.68	1.957	0.597
S6	30	2.5	10	3.00	1.279	0.449

**Table 3:** Probable maximum precipitation (PMP) estimation of Batu dam.

Method	PMP in mm for Various Durations							
	1-h	3-h	6-h	12-h	1-Day	2-Day	3-Day	5-Day
Statistical–Hershfield’s Method (Derived Km)	104.38	182.62	197.26	187.02	215.68	305.63	329.90	437.27
Statistical–NAHRIM Method (K_m_ Envelope Curve)	179.31	261.91	280.43	309.01	398.42	549.59	557.78	613.27

**Table 4:** Designed inflow and designed discharge capacities based on PMF values.

Dam	Designed Inflow (m^3^/s)	PMF (m^3^/s)	Designed Discharge Capacities (m^3^/s)	Revised Spillway Capacities (m^3^/s)	Reservoir Level for Designed Inflow (m)	Simulated Reservoir Level (m)
Batu	492.7	522.7	234.2	200.6	108.0	107.4

## Data Availability

The data adopted in this study are available at the National Hydrological Network Management System (SPRHiN). Any interested person(s) could get it from the website at http://sprhin.water.gov.my/bin/borang/login.cfm (accessed on 18 May 2022).
